# Dominance of Vaccine‐Specific *Chlamydia pecorum ompA* Genotypes in Koalas From North‐Eastern Australia

**DOI:** 10.1002/ece3.70973

**Published:** 2025-03-07

**Authors:** Derek S. Sarovich, Martina Jelocnik, Praphaporn Stewart, Nina M. Pollak, Jessie S. F. Wong, Charlotte Kunesh, Chioma Ojiako, Jon Hanger, Amber Gillett, Ludovica Valenza, Timothy Portas, Jodie Wakeman, Peter Timms, Samuel Phillips

**Affiliations:** ^1^ Centre for Bioinnovation University of the Sunshine Coast Sippy Downs Queensland Australia; ^2^ School of Science, Technology and Engineering University of the Sunshine Coast Sippy Downs Queensland Australia; ^3^ Princeton University Princeton New Jersey USA; ^4^ Endeavour Veterinary Ecology Toorbul Queensland Australia; ^5^ Australia Zoo Wildlife Hospital Beerwah Queensland Australia; ^6^ RSPCA Queensland Wildlife Hospital Wacol Queensland Australia; ^7^ Friends of the Koala Wildlife Hospital Lismore New South Wales Australia

**Keywords:** *Chlamydia pecorum*, genotyping, immunology, koala, MOMP, *omp*A, vaccine

## Abstract

*Chlamydia pecorum* is a significant cause of morbidity and mortality in koalas and a major contributor to population decline. Due to its crucial role in vaccine development and use as a strain typing tool, multiple studies have investigated the prevalence and diversity of the *C. pecorum* outer membrane protein A (MOMP), encoded by *omp*A. This prior work has shown that *omp*A genotypes vary across geographical regions, with multiple genotypes identified across Eastern Australia. Despite these earlier studies, our understanding of the diversity and distribution of MOMP remains incomplete. Here, we aimed to assess the geographical distribution, diversity, and temporal stability of the *C. pecorum* *omp*A across wild koalas infected with *Chlamydia* in north‐eastern Australia. Full‐length *omp*A sequences were generated from 226 samples collected from 173 unique animals from multiple koala populations over a 4‐year time span. *C. pecorum omp*A genotypes F and G were the most common and were identified across all geographical regions. Genotype A was the next most common but mostly restricted to the South Brisbane region. In addition to two novel genotypes, rare genotypes, including livestock‐associated types E58 and L1, and koala‐associated H and I were sporadically identified. Comparison with historical *omp*A genotypes showed that the distribution of genotypes has remained stable over the past decade, suggesting limited selective pressure despite ongoing vaccination trials and management programmes. The stability of genotypes coupled with the identification of novel and livestock‐associated genotypes underscores the importance of continued surveillance to inform future vaccine development and conservation strategies for koalas.

## Graphical abstract

The comprehensive dataset, including full‐length *ompA* sequences from over 220 samples, provides a unique look at the geographical spread and temporal stability of *Chlamydia pecorum* . Our analysis not only underscores the prevalence of vaccine‐specific genotypes but also highlights the emergence of novel genotypes and their potential implications for vaccine effectiveness. Such insights are crucial for devising strategies to manage and conserve affected koala populations.

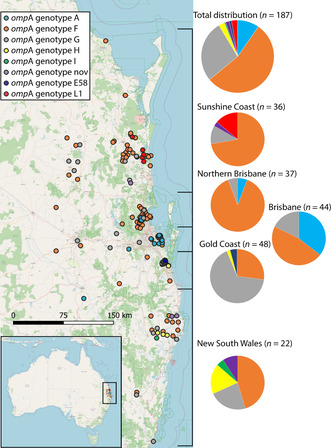

## 1 Introduction

*Chlamydia pecorum* is an obligate intracellular bacterium that infects various animal hosts, including koalas ( *Phascolarctos cinereus* ) (Quigley and Timms 2020). It is a significant pathogen in this species, causing severe health issues such as conjunctivitis, cystitis and reproductive disease, which can lead to blindness, urinary incontinence and infertility (Polkinghorne et al. 2013). These infections are a significant factor in the decline of koala populations, making the control and treatment of *C. pecorum* a high priority for conservation efforts (Gonzalez‐Astudillo et al. 2017).

Over the past 15 years, significant efforts have been made to develop a Chlamydia vaccine for koalas. Over a dozen separate trials have been conducted in South‐East Queensland, exploring various antigens, adjuvants, delivery methods and the robustness of immune responses (Desclozeaux, Robbins, et al. 2017; Khan, Desclozeaux, et al. 2016; Khan, Polkinghorne, et al. 2016; Quigley et al. 2023; Waugh et al. 2016). The majority of these trials have focused on using recombinant *C. pecorum* major outer membrane protein (MOMP), encoded by *omp*A, as the antigenic target and involving three distinct genotypes (MOMP A, F and G). Collectively, these trials have demonstrated that MOMP‐based vaccines can induce a strong immune response with only a single dose. Most recently, the effectiveness of the MOMP‐based vaccine was evaluated in a managed koala population over a 10‐year time span. This study demonstrated that the vaccine reduces both the incidence of chlamydial disease, moving the median age of disease development out to 3 years, and decreased mortality by 64% (Phillips et al. 2024).

The *omp*A gene has been used across multiple studies as a strain typing tool to assess chlamydial diversity in koala populations (Kollipara et al. 2013; Legione, Patterson, et al. 2016; Nyari et al. 2017; Wedrowicz et al. 2018). Together, these studies have identified 15 *omp*A genotypes, separated into two clades and composed of genotypes A through O. Some geographical trends have been identified among these genotypes, with A, E', F, F′, H, J and K more prevalent in north‐east Australia and genotypes B, C, G, I, L, M, N and O more prevalent in the south, although this separation is not absolute, with genotypes F, F′, G and M being detected in both regions (Quigley and Timms 2020; Desclozeaux, Robbins, et al. 2017). Interestingly, multiple studies have identified overlap with *C. pecorum* *omp*A genotypes infecting cattle, sheep and other livestock, suggesting that *C. pecorum* strains move between these species (Legione, Patterson, et al. 2016; Hagenbuch et al. 2024; Kasimov et al. 2022; Robbins et al. 2020).

In the current study, we aimed to better understand the genetic diversity, distribution and temporal stability of *omp*A within the *C. pecorum* population infecting koalas in South‐East Queensland and northern New South Wales (NSW). A thorough understanding of *C. pecorum* infection dynamics and *omp*A diversity will assist future vaccine design and other conservation efforts.

## 2 Methods

### 2.1 Study Sites and Sample Collection

Swabs collected during screening of wild koalas from November 2020 to March 2024 from four wildlife hospitals (Australia Zoo Wildlife Hospital [AZWH], Beerwah, Queensland, Australia; The Royal Society for the Prevention of Cruelty to Animals [RSPCA], Wacol, Queensland, Australia; Endeavour Veterinarian Ecology [EVE], Toorbul, Queensland and Friends of the Koala Inc. [FoK], Lismore, NSW, Australia) were utilised. Where possible, swabs from multiple anatomical sites were obtained and screened for *C. pecorum* (Hulse et al. 2019; Jelocnik et al. 2017). Metadata, including the date, location and clinical manifestations, were recorded for each sampled koala.

Samples were obtained from 173 distinct animals across north‐eastern Australia, spanning from Coffs Harbour to Bundaberg, with sampling occurring from November 2020 to March 2024. Animal demographics, including age, weight, sex, body condition score, anatomical sampling site and geographical location, are included in Table S1. Sampling density varied across regions, with the highest density obtained in the Gold Coast, yielding 64 samples from 37 distinct animals. The Brisbane region, including Redland and western regions, yielded 58 samples from 43 distinct animals. The North Brisbane (Moreton Bay) region yielded 41 sequences from 36 distinct animals. The Sunshine Coast region, including Gympie, Fraser Coast and Bundaberg, yielded 39 samples from 36 distinct animals. Sampling from NSW (including Byron, Lismore, Ballina, Richmond Valley, Kyogle, Clarence Valley and Coffs Harbour) yielded 29 samples from 21 distinct animals (Table S1).

To avoid redundancy and sampling bias when comparing *omp*A genotype diversity, both geographically and longitudinally, we removed identical *omp*A genotypes when identified from different anatomical sites within the same animal (Table S1). Samples obtained from different anatomical sites that contained distinct *omp*A genotypes were included as unique samples (Table S1). Additionally, multiple longitudinal samples obtained from the same animal within a sampling interval of < 3 months have been removed from geographical and longitudinal analyses, resulting in a unique sample dataset of *n* = 187. All generated sequence data (*n* = 226) were used for phylogenetic and evolutionary analyses.

### 2.2 Full Length *ompA* Sequencing

All swabs were placed in 500 μL sterile phosphate buffered saline, vortexed and 200 μL used for DNA extraction using the QiaAMP DNA mini kit according to the manufacturer's instructions (Qiagen, Australia). In order to confirm the presence of koala and *C. pecorum* DNA, samples were screened in duplicate with species‐specific SYBR Green‐based qPCR assays targeting the koala beta actin gene and a conserved *C. pecorum* target, as previously described (Hulse et al. 2019; Shojima et al. 2013). All positive samples were subjected to full‐length *omp*A gene amplification using conventional PCR with the following primers: *omp*A‐F (5′‐AAGCATAATCTTAGAGGTGAG‐3′) and *omp*A‐R (5′‐CTGTTAGAATCTGCATTGAGC‐3′) and PCR cycling protocol, as previously described (Islam et al. 2019). PCR products were visualised on a 1.5% agarose gel and sequenced through Macrogen Inc. (South Korea). Chromatograms were visualised for quality prior to merging forward and reverse reads, followed by manual alignment, inspection and curation in Geneious Prime v. 2024.0.4 (Kearse et al. 2012).

### 2.3 *omp*A Genotype Comparisons

The prevalence of *omp*A genotypes, in both managed (northern Brisbane) and unmanaged (Gold Coast) populations, was compared to historical data and tested for significant differences in distribution using Pearson's chi‐square test. All *omp*A defined types from 2013 were derived from full or partial VD3–VD4 *omp*A gene sequences, as previously described (Kollipara et al. 2013; Nyari et al. 2017; Robbins et al. 2020). A Wilcoxon rank‐sum test was used to identify associations with animal demographics and *omp*A genotypes when testing continuous variables. A chi‐square test was used when testing for an association between *omp*A genotype and categorical variables.

### 2.4 *ompA* Sequence Analysis

Nucleotide sequences were translated into proteins using translation tools from the first open reading frame implemented in Geneious Prime. MOMP sequences were aligned using MUSCLE. *omp*A genotype was assigned to the closest known genotype using BLAST. Fast unconstrained Bayesian AppRoximation (FUBAR) (Murrell et al. 2013) was used to investigate selection pressure at each site in the MOMP protein. Sequences are available in Data S1.

### 2.5 Ethics

This study was approved by the University of the Sunshine Coast (UniSC) Animal Ethics Committee (ANA20179) and by the Queensland Government (Scientific Purposes Permits, WA0032319).

## 3 Results

### 3.1 Genotyping of *omp*A Shows Predominance of Genotypes A, F and G Across Eastern Australia

**FIGURE 1:**
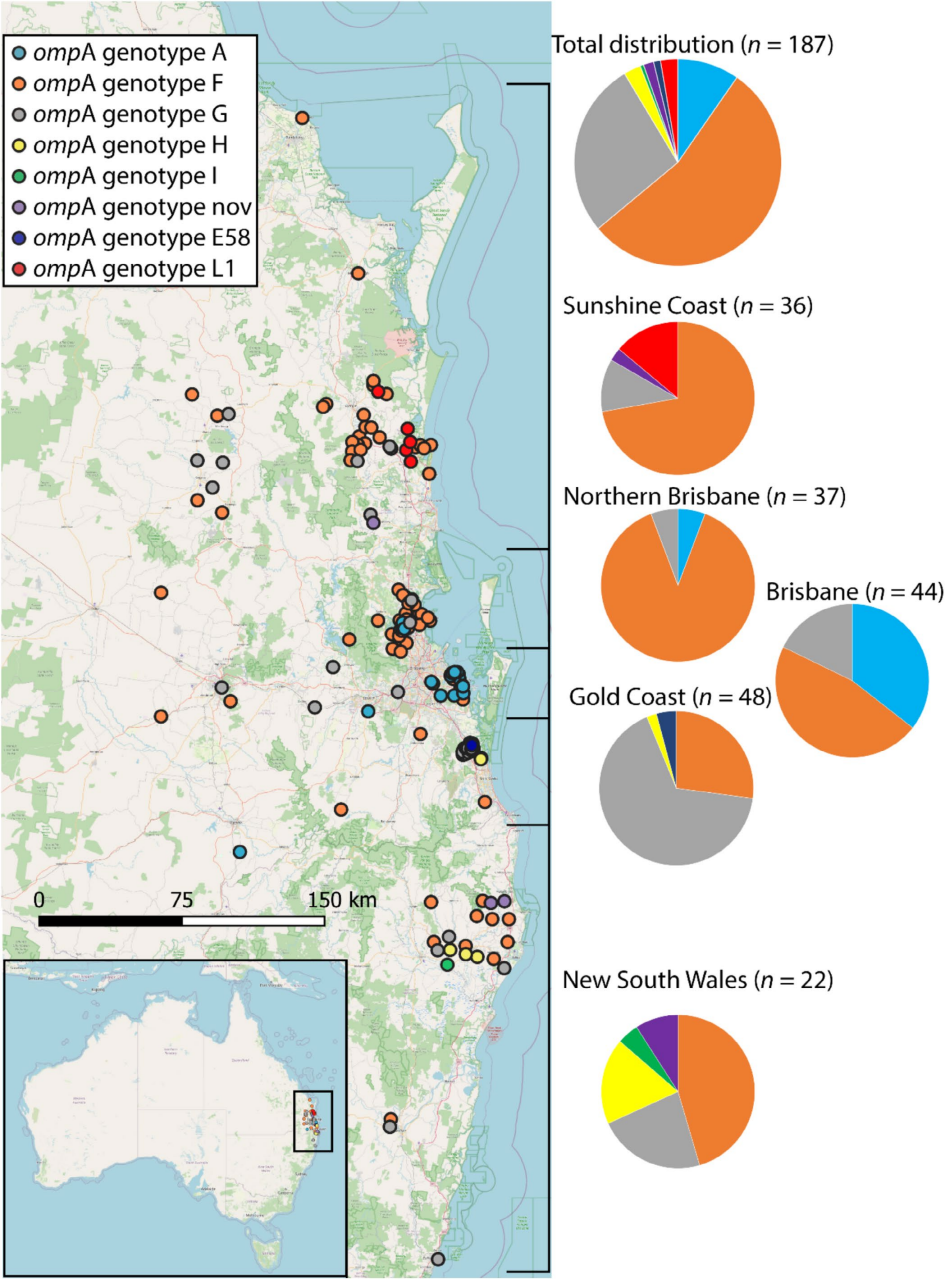
Distribution of *omp*A genotypes across South‐East Queensland and northern New South Wales, Australia. Different *omp*A genotypes are represented by different coloured circles. The *omp*A genotypes F and G were common and identified across all regions. The *omp*A genotype A was the next most common but geographically restricted to the Brisbane and north Brisbane/western regions. Additional *omp*A genotypes were found sporadically across multiple regions with some geographical biases.

The distribution of *omp*A genotypes varied considerably among the multiple geographical regions across north‐eastern Australia (Figure 1). Overall, *omp*A genotype F was the most prevalent, found in 54.0% of unique samples (*n* = 101) and with particular dominance in the northern Brisbane (Moreton Bay) and Sunshine Coast regions (Table 1; Table S1). Genotype G was also frequently identified across all regions, found in 26.7% of unique samples (*n* = 50). *omp*A genotype A was the next most common at 10.2% (*n* = 19), frequently identified in the Brisbane region, with sporadic identification in the northern Brisbane region. Less common genotypes (i.e., the livestock associated genotypes L1 (Rohner et al. 2021), and E58, and the koala associated genotypes H and I) showed sporadic presence across multiple regions, ranging from 0.5% to 2.7% prevalence (Table 1).

**TABLE 1:** Distribution of *ompA* genotypes identified in each region.

State (region/s)	Genotype counts (%)								Total
	A	E58	F (group)	G	H	I	L1	Nov.	
Queensland (Sunshine Coast, Gympie, Fraser Coast, Bundaberg)	0	0	26 (72.2)	3 (8.3)	0	0	5 (13.9)	2 (5.6)	36
Queensland (northern Brisbane [Moreton Bay])	2 (5.4)	0	33 (89.2)	2 (5.4)	0	0	0	0	37
Queensland (Brisbane, Redland, Western regions)	17 (38.6)	0	19 (43.2)	8 (18.2)	0	0	0	0	44
Queensland (Gold Coast)	0	2 (4.2)	13 (27.1)	32 (66.7)	1 (2.1)	0	0	0	48
New South Wales (Byron, Lismore, Ballina, Richmond Valley, Kyogle, Clarence Valley, Coffs Harbour)	0	0	10 (45.5)	5 (22.7)	4 (18.2)	1 (4.5)	0	2 (9.1)	22
Totals	19 (10.2)	2 (1.1)	101 (54.0)	50 (26.7)	5 (2.7)	1 (0.5)	5 (2.7)	4 (2.1)	187

*Note:* Longitudinal sampling occurred from November 2020 to March 2024. Identical *ompA* genotypes, identified from different anatomical sites from the same animal, have been removed. Multiple longitudinal samples obtained from the same animal within a sampling interval of < 3 months have been removed.

Abbreviation: Nov, novel.

The Sunshine Coast region (*n* = 36), including Gympie, Fraser Coast and Bundaberg, showed a high prevalence of genotype F (72.2%), followed by genotype L1 and G at 13.9% and 8.3%, respectively. Additionally, a novel *omp*A genotype was identified and found in two animals (Figure 1; Table 1). Genotype F was overwhelmingly predominant in the northern Brisbane region, representing 89.2% of samples. Genotypes A and G were minimally present at 5.4% each. In the Brisbane, Redland, Western Downs and Toowoomba regions, there was a significant presence of *omp*A genotypes A and F at 38.6% and 43.2% prevalence, respectively. Genotype G was identified in 18.2% of unique samples (Figure 1; Table 1). The *omp*A genotype distribution in the Gold Coast region (*n* = 48) showed a predominance of *omp*A genotype G (66.7%), followed by genotype F (27.1%), genotype E58 (4.2%) and H (2.1%).

Due to low sample numbers, the northern New South Wales regions were grouped together and contained the Byron, Lismore, Ballina, Richmond Valley, Kyogle, Clarence Valley, and Coffs Harbour regions (*n* = 22). The distribution of *omp*A genotypes was more varied across these regions, with genotype F leading at 45.5%, followed by genotypes G and H at 22.7% and 18.2%, respectively. Two samples (9.1%) contained a novel *omp*A genotype, and one sample was identified as *omp*A genotype I (Figure 1; Table 1).

### 3.2 Comparison of Prior *omp*A Genotype Distribution Shows Stability Over Time

We compared our *omp*A genotype prevalence from the managed koala population in the northern Brisbane region (*n* = 37 unique samples) with prior work assessing *omp*A diversity to identify any potential changes in *omp*A distribution over time. Nyari et al. (2017) and Robbins et al. (2020) sequenced *omp*A from 75 koalas in the same koala population between 2013 and 2014 and identified a predominance of *omp*A genotype F, followed by genotype G and A. When compared to the distribution in the current work, no statistically significant changes were identified (*p*‐value = 0.35), despite these samples being collected 10 years prior (Table S2). Historical data were also available from the unmanaged koala population in the gold coast regions, sampled in 2013 (Kollipara et al. 2013). Prior analysis showed a predominance of *omp*A genotype G (13/14 samples [92.8%]), with a low proportion of genotype A (1/14 samples [7%]). No significant difference was identified between this historical distribution and the current *omp*A distribution (Table S2).

### 3.3 Full Length *ompA* Sequencing Identified Minimal Amino Acid Diversity Within MOMP Genotypes, Novel Genotypes, and Livestock‐Associated Genotypes

**FIGURE 2:**
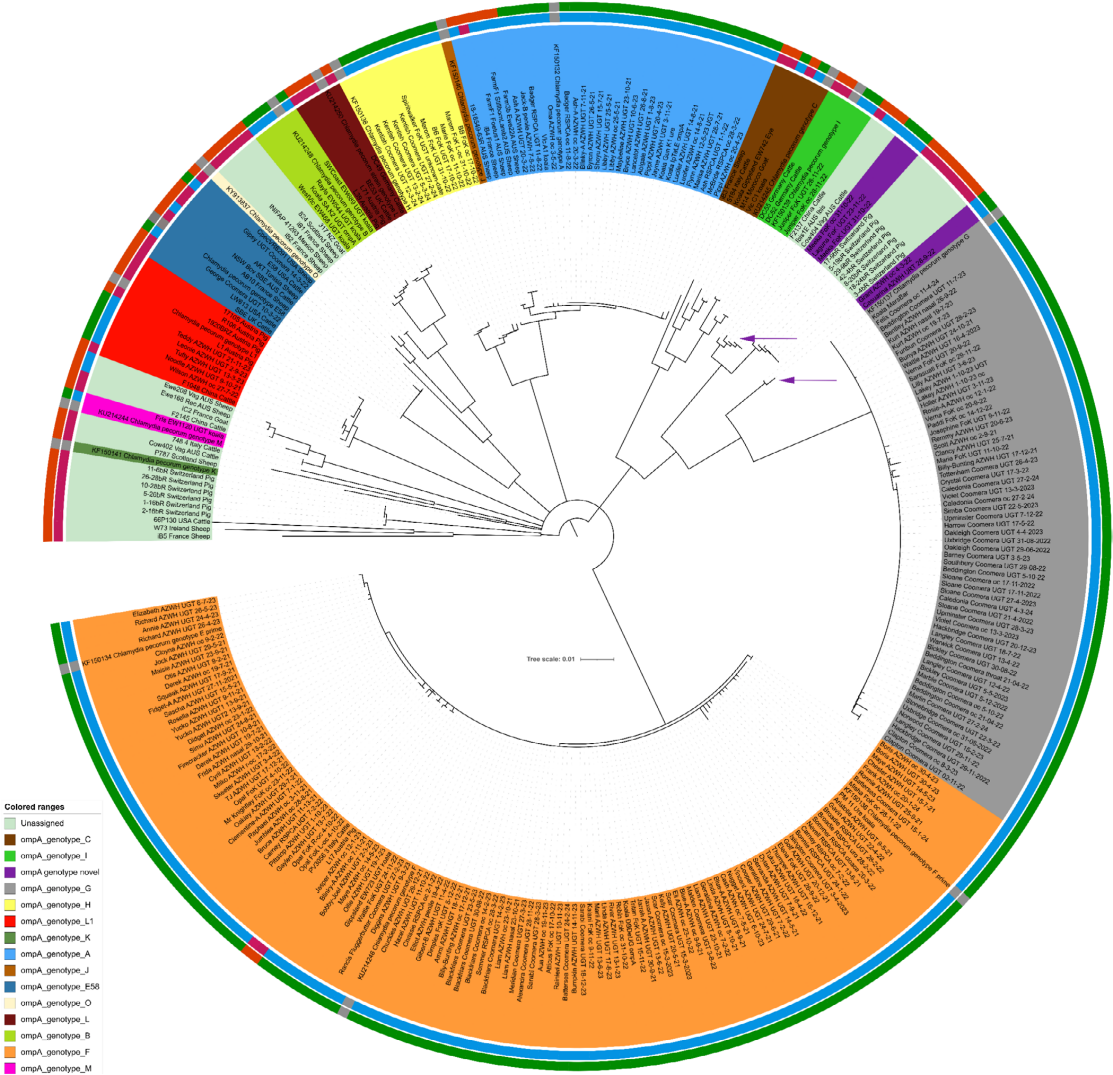
Phylogenetic relatedness tree of 295 *Chlamydia pecorum* *omp*A sequences and associated geographical source, animal host and genotype. Full‐length *omp*A sequences were generated from 226 samples in the current study. The most common *omp*A genotypes identified were F group (54%), followed by G (26.7%) and A (10.2%). Novel genotypes identified in this study are indicated by purple arrows. Outer ring shading denotes geographical origin; green = Australian, orange = non‐Australian, grey = reference sequence. Inner ring denotes animal host; blue = koala, red = other, grey = reference sequence. *omp*A genotypes E′ and F′ are assigned to genotype F due to limited diversity among these genotypes.

A neighbour‐joining phylogenetic tree was created using all 226 *omp*A nucleotide sequences generated in this study and compared against publicly available *C. pecorum omp*A derived from both koala and non‐koala hosts (Figure 2). Importantly, to identify fine‐scale resolution, we chose to include all *omp*A sequences even if derived from the same animal or separated by < 3 months.

First, limited amino acid diversity (> 98% similarity) was observed among MOMP genotypes E′, F′ and F, regardless of geographical source or animal origin (Figure 2). Limited diversity was also noted in the other vaccine MOMP genotypes A and G, with > 99% amino acid similarity within these genotypes. The limited genetic diversity within these groups, in addition to their frequent identification, meant that 65% of samples matched with 100% amino acid similarity to one of the MOMP genotypes included in the vaccine (i.e., A, F, and G), and 100% of samples matched with > 95% amino acid similarity (Table S3).

Second, as mentioned prior, two novel *omp*A genotypes were identified during the initial genotype prevalence assessment. Novel genotype 1 was identified in two koalas from northern New South Wales, and novel genotype 2 was identified in two koalas from the Sunshine Coast region (Figure 2; Table S1). Novel genotype 1 was most closely related to the *omp*A genotype G (88% amino acid identity), and novel genotype 2 was most closely related to the *omp*A genotype F (86% amino acid identity). Although infrequent, livestock‐associated *omp*A genotypes, L1 (Rohner et al. 2021) and E58, were identified in multiple geographical locations (Figure 1). Genotype L1 was identified in five animals, all sampled in the Sunshine Coast region, and *omp*A genotype E58 was identified in three animals from the Gold Coast region.

### 3.4 Associations of *omp*A Genotype and Animal Demographics

Association testing was performed to investigate the correlation between *omp*A genotype and demographic information. No significant associations were identified with *omp*A genotype and koala age, weight, sex, anatomical site or season of admission (Table S4). Unsurprisingly, significant associations were identified with most *omp*A genotypes and geographic location (Table S4). No significant association was identified for *omp*A genotype E58, likely due to low sample numbers of this genotype.

### 3.5 Amino Acid Changes in MOMP Are Indicative of Both Positive and Negative Selection Pressure

FUBAR (Murrell et al. 2013) was used to identify any signals of pervasive selection. Nineteen amino acids (sites) were identified as undergoing pervasive positive or diversifying selection, with eight sites (18, 92, 96, 259, 261, 332, 333 and 337) showing strong or very strong evidence for positive selection. Seven of these sites were located in predicted outer membrane domains, with the last site located in the leader sequence. In contrast, a large number of sites (180 amino acids) showed signs of pervasive negative or purifying selection (Table S5; Figure S1).

## 4 Discussion

Our study provides a comprehensive analysis of *C. pecorum* *omp*A genotype distribution across South‐East Queensland, which encompasses one of the largest concentrations of koala populations in Australia, along with additional sampling from northern New South Wales. Our work highlights the dominance of *omp*A genotypes F and G, which were identified across all sampling locations. Additionally, we show that the *omp*A genotype distribution has remained stable in both managed and unmanaged koala populations across a 10‐year time span. Notably, two novel *omp*A genotypes, not previously identified in koalas, were found.

The dominance of *omp*A genotype F, particularly in the northern Brisbane (Moreton Bay) and Sunshine Coast regions (Figure 1), is consistent with previous studies (Nyari et al. 2017; Robbins et al. 2020). The northern Brisbane region has been subject to intensive veterinary management efforts, including the implementation of *C. pecorum* vaccination programmes aimed at reducing *C. pecorum* morbidity and mortality (Phillips et al. 2024; Nyari et al. 2017). Despite these interventions, the overall distribution of *omp*A genotypes has remained stable, suggesting that intensive veterinary management has exerted limited selective pressure on *omp*A diversity. In contrast to this managed koala population, the Gold Coast region has had no major human management interference across a similar timeframe (Kollipara et al. 2013). Despite these differences in population management, *omp*A diversity appears temporally consistent in both the managed and unmanaged populations, suggesting that natural population dynamics and low selective pressures have resulted in a stable distribution of these genotypes over time, despite Chlamydia vaccine rollout in the managed population (Phillips et al. 2024). While the overall *omp*A diversity remained consistent, our work identified a low proportion of genotypes not previously identified. Given the low number of historical samples, it is impossible to determine whether these genotypes were not detected in the prior work due to the limited sampling or if they are a more recent introduction into these areas.

The regional diversity of *C. pecorum* *omp*A genotypes across Australia underscores the influence of geographical and ecological factors on genotype distribution. In contrast to the current work where we noted a high prevalence of *omp*A genotypes F and G in South‐East Queensland, wider geographical sampling of southern regions (e.g., Victoria and South Australia) has reported prevalence of distinct *omp*A genotypes, such as L, B and C, which were absent in South‐East Queensland. Despite these differences, overlaps were observed with nearby regions (e.g., New South Wales), where genotype F also predominated alongside occasional detections of shared genotypes such as G. This interplay of localised unique distribution and broader overlap illustrates the complex dynamics shaping *C. pecorum* diversity across Australia.

Prior work has investigated *omp*A genotypes and their distribution among livestock (Hagenbuch et al. 2024; Jelocnik et al. 2023), from multiple locations globally (Legione, Patterson, et al. 2016; Legione, Amery‐Gale, et al. 2016; Hagenbuch et al. 2024; Rohner et al. 2021; Limon‐Gonzalez et al. 2022; Mohamad et al. 2014; Fernandez et al. 2024; White et al. 2021). In the current study, we identified the livestock‐associated *omp*A genotypes L1 and E58 in low numbers (Figures 1 and 2). The *omp*A genotype L1, originally identified in a chlamydial outbreak in an Austrian pig farm in 1969 (Kolbl 1969; Kolbl et al. 1970), was found in five animals in the Sunshine Coast region. The *omp*A L1 identified in our study differs from the historical *omp*A L1 by four non‐synonymous mutations, with all changes occurring in the hypervariable regions of MOMP. Similarly, *omp*A genotype E58 was also identified, which has been previously found in strains infecting cattle and sheep in Australia and elsewhere (Jelocnik et al. 2023). Efforts to address *C. pecorum* infections in livestock have included the investigation of prototype recombinant vaccines (Desclozeaux, Jelocnik, et al. 2017), which have demonstrated safety and immunogenicity, particularly in lambs. These studies highlight the recognition of *C. pecorum* as a significant pathogen in livestock, with ongoing efforts to mitigate its impact through immunological interventions (Bommana et al. 2017). As noted prior, the presence of livestock‐associated *omp*A genotypes and the identification of rare and common *omp*A genotypes in both koalas and livestock raise questions about potential animal‐to‐animal transmission or shared environmental reservoirs (Hagenbuch et al. 2024; Jelocnik et al. 2023). It should be noted that the analyses conducted in this study do not conclude that the ‘infecting strain’ necessarily belonged to a non‐koala, but rather that the single *omp*A gene was similar to non‐koala *omp*A sequences. Although our study is limited to a single gene, this close relatedness suggests a recent common ancestor or recombination of the *omp*A alleles. Further research using higher resolution typing methods, including whole‐genome sequencing, ideally with additional sampling, is needed to address these questions.

We observed limited amino acid diversity within MOMP genotypes E′, F′ and F, which strongly suggests that these clades should be reclassified as a single group (Figure 2). In support of this reclassification, a similar level of diversity was observed within other major genotype groups (Figure 2). Despite the limited diversity within *omp*A genotypes, we identified a strong signal of positive selection within *omp*A; 19 amino acids were identified as being under strong or very strong positive evolutionary pressure, with most amino acids found in outer membrane variable domains. In concordance with prior work (Phillips et al. 2020), these regions likely play a crucial role in the immune response and are likely essential for vaccine efficacy. Interestingly, there was a single site in the leader sequence that showed evidence of positive selection, suggesting that mutations in this site may confer advantages in terms of protein folding, stability or membrane insertion, contributing to fitness under specific environmental conditions. In contrast to the handful of amino acids under positive selection, a large number of amino acids (180 sites) were identified as being under purifying selection. The current vaccine (Phillips et al. 2024), which includes recombinant MOMP genotypes A, F and G, will likely provide broad coverage across South‐East Queensland, given the limited diversity within these genotypes and a high (> 95%) similarity between all *omp*A genotypes. This predicted efficacy is further supported by our recent work, which showed that vaccinated koalas had a significantly lower incidence of chlamydial disease and a 64% reduction in associated mortality (Phillips et al. 2024). However, the presence of novel or livestock‐associated genotypes may pose challenges to vaccine efficacy, although some cross‐protection is expected (Sangewar et al. 2020).

This study has several acknowledged limitations. The sampling in some geographical regions, both contemporarily for northern New South Wales and historically for the Gold Coast and northern Brisbane region, was limited and underpowered our ability to identify *omp*A genotype changes over time, particularly for rare genotypes. Additionally, our focus on the *omp*A gene alone limited our ability to precisely determine the origin of the livestock‐associated *omp*A genotypes, and due to the lowered sensitivity of the full‐length *omp*A PCR, we chose not to look at infection prevalence, which has been covered extensively elsewhere (Quigley and Timms 2020). We acknowledge that it would be interesting to investigate the correlation between animal health and *omp*A genotype; however, limitations in data capture prevent a robust statistical analysis across multiple veterinary hospitals, and the focus on a single locus neglects the effects of other immunogenic or virulence loci. Lastly, no contemporary koala samples were included from other regions of Australia, such as northern, central and western Queensland, Victoria and South Australia, limiting the generalisability of our findings to these areas.

Our study provides valuable insights into the *omp*A genotype distribution of *C. pecorum* in koala populations across South‐East Queensland and northern New South Wales. The stability of *omp*A genotypes over time, combined with the identification of novel genotypes and livestock‐associated strains, underscores the need for ongoing surveillance and research to inform vaccine development and conservation efforts for koalas. Future studies should aim to include broader geographic sampling and consider whole‐genome analyses to fully understand the genetic diversity and evolutionary dynamics of *C. pecorum* in koalas.

## Author Contributions

**Derek S. Sarovich:** data curation (lead), formal analysis (lead), investigation (equal), methodology (lead), resources (supporting), supervision (supporting), visualization (lead), writing – original draft (lead), writing – review and editing (lead). **Martina Jelocnik:** conceptualization (supporting), data curation (supporting), methodology (supporting), resources (supporting), supervision (supporting), writing – review and editing (supporting). **Praphaporn Stewart:** data curation (supporting), formal analysis (supporting), methodology (supporting), resources (supporting), writing – review and editing (supporting). **Nina Pollak:** project administration (supporting), writing – review and editing (supporting). **Jessie Wong:** formal analysis (supporting), investigation (supporting), methodology (supporting), resources (supporting), validation (supporting). **Charlotte Kunesh:** data curation (supporting), investigation (supporting). **Chioma Ojiako:** methodology (supporting), resources (supporting). **Jon Hanger:** data curation (supporting), methodology (supporting), resources (supporting), writing – review and editing (supporting). **Amber Gillett:** data curation (supporting), methodology (supporting), resources (supporting), writing – review and editing (equal). **Ludovica Valenza:** data curation (supporting), methodology (supporting), resources (supporting). **Timothy Portas:** data curation (supporting), methodology (supporting), resources (supporting). **Jodie Wakeman:** data curation (supporting), methodology (supporting), resources (supporting). **Peter Timms:** conceptualization (equal), funding acquisition (equal), investigation (equal), methodology (equal), project administration (equal), resources (equal), supervision (equal), writing – review and editing (equal). **Samuel Phillips:** conceptualization (equal), data curation (equal), funding acquisition (equal), methodology (equal), project administration (equal), resources (equal), supervision (equal), writing – review and editing (equal).

## Conflicts of Interest

The authors declare no conflicts of interest.

## Supporting information


**Figure S1.** Fast unconstrained Bayesian AppRoximation (FUBAR) estimation of evolutionary pressure across the MOMP protein. Nineteen amino acids sites show evidence of positive selection (positive values along the *Y*‐axis), suggesting that these sites are immunologically significant. Red arrows point to six sites identified to be under very strong positive selection, with a Bayes factor > 100. One hundred and eighty amino acids were identified as being under purifying selection pressure (negative values along the *Y*‐axis). Coloured bars denote different protein regions, estimated in a prior study (Phillips et al. 2020). Individual sites are coloured according to their Bayes factor score for positive selection. *Y*‐axis indicates strength of positive (above zero) or negative (below zero) selection pressures. BB, beta‐barrel; IM, inner membrane; OM, outer‐membrane; VD, variable domain.

**Table S1.** *ompA* genotypes and associated animal metadata.
**Table S2.** Historical *omp*A genotype prevalence in the northern Brisbane and Gold Coast regions in comparison to the current distribution.
**Table S3.** Amino acid identity for all samples versus all samples across the full‐length MOMP.
**Table S4.** *ompA* genotype and association with animal demographics.
**Table S5.** Full FUBAR output across the entire *omp*A gene.

**Data S1.** ompA sequences used in this study.

## Data Availability

All data generated or analysed during this study are openly accessible and included as Supporting Information. Metadata are provided alongside each dataset to ensure transparency and reusability, following FAIR (Findable, Accessible, Interoperable and Reusable) principles.
