# Supplementary figures and images for: Dominance of Vaccine‐Specific Chlamydia pecorum ompA Genotypes in Koalas From North‐Eastern Australia
Source: Ecol Evol. 2025 Mar 7;15(3):e70973. doi: 10.1002/ece3.70973 (PMC11885949; doi:10.1002/ece3.70973)

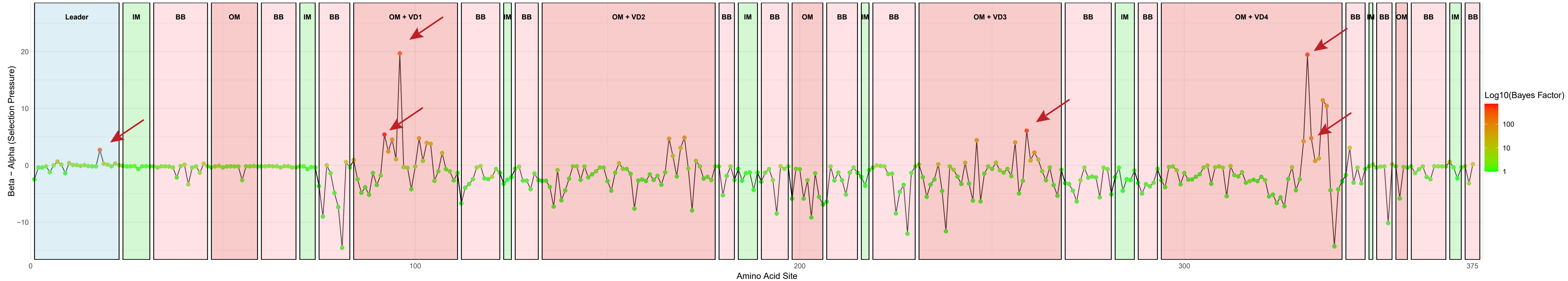

Supplement: Supplementary file 1 — Figure S1. Fast unconstrained Bayesian AppRoximation (FUBAR) estimation of evolutionary pressure across the MOMP protein. Nineteen amino acids sites show evidence of positive selection (positive values along the Y‐axis), suggesting that these sites are immunologically significant. Red arrows point to six sites identified to be under very strong positive selection, with a Bayes factor > 100. One hundred and eighty amino acids were identified as being under purifying selection pressure (negative values along the Y‐axis). Coloured bars denote different protein regions, estimated in a prior study (Phillips et al. 2020). Individual sites are coloured according to their Bayes factor score for positive selection. Y‐axis indicates strength of positive (above zero) or negative (below zero) selection pressures. BB, beta‐barrel; IM, inner membrane; OM, outer‐membrane; VD, variable domain. [file ECE3-15-e70973-s001.pdf]
